# The impact of anxiety and depression levels on the Big Five personality traits

**DOI:** 10.1371/journal.pone.0321373

**Published:** 2025-07-24

**Authors:** Fatma Tuygar Okutucu, Hacer Akgul Ceyhun

**Affiliations:** Department of Psychiatry, Ataturk University Medical Faculty, Erzurum, Turkey; Aalborg University, DENMARK

## Abstract

**Background:**

The prevalence of stress, anxiety, depression and burnout is quite high in medical students, and they differ in terms of personality traits and self-perception. The Big Five Model has embraced the idea of five basic personality traits: neuroticism, extroversion, conscientiousness, agreeableness, openness, and personality perceptions may be affected by stress. We aimed to test whether there are changes in the Five-Factor Model personality traits of medical students in response to anxiety and depression levels.

**Methods:**

We evaluated the prevalence of anxiety and depressive symptoms among medical students. Beck Anxiety Inventory(BAI) and Beck Depression Inventory(BDI) were used respectively. The Five Factor Personality Inventory(FFPI) was used to evaluate the personality traits and to investigate their associations with anxiety and depressive symptoms. A self-administered questionnaire was sent to 900 students via e-mail and 733 data were analyzed.

**Results:**

Anxiety (n=233, 31.8%) and depression (n=184, 25.1%) were found to be high in medical students. History of psychiatric illness and suicide were significantly high in those with high anxiety and depression levels. In the correlation analysis, increasing anxiety and depression levels were positively correlated with neuroticism and negatively correlated with extraversion, conscientiousness, agreeableness, and openness. In the linear regression analysis, it was seen that both depression and anxiety positively influenced neuroticism and anxiety negatively influenced openness.

**Conclusion:**

Depression and anxiety symptoms are prevalent among medical students and had an impact on their personality traits. Depression had an impact on neuroticism, and anxiety had an impact on openness. However, these results should be confirmed with prospective cohort studies.

## Introduction

The prevalence of stress, anxiety, depression and burnout is quite high among medical students, and they differ in terms of personality traits and self-perception [1]. Medicine is one of the most sought-after university degree courses and mental disorders are more common among medical students than is generally acknowledged. Both depression and burnout and anxiety are significant issues that are frequently addressed in medical students. Some factors that may affect the mental health of this population include high academic workload, proximity to patient suffering, limited social life, poor family life, lack of sleep and inconsistent and distant romantic relationships. These factors can be seen as obstacles that test medical students during their training phase and set them apart from other university students [2].

While personality traits are generally considered stable, new theories suggest that they may change due to life events and psychosocial processes. In particular, state effects of anxiety and depression on personality traits have been observed, warranting further investigation of their effects on students’ well-being and academic performance. Medical students are particularly vulnerable to psychological distress due to their demanding training, making them an ideal population to study the impact of anxiety and depression on personality traits [3,4]. The Big Five traits are important because they predict a variety of outcomes, including academic performance and psychological resilience [5].

Studies have shown that traits such as empathy and other interpersonal skills may deteriorate in medical students during rigorous medical training [6,7]. It can be said that the medical student population has a homogeneous distribution in many respects. Their average age is similar, they generally come from high socio-economic backgrounds and their education levels are high. They can be generally described as intelligent, conscientious, highly motivated, ambitious, and perfectionist people [8]. However, personality traits are influenced by other traits and depend on different contexts [9,10]. It is necessary to determine how it can contribute to increasing the well-being of students and developing personality traits that can affect their resilience. This type of well-being not only benefits them as students but can also help them understand how to improve the health of their patients when they become physicians [11].

Personality emerges from characteristic thinking, feeling, and behavior features and is a way of classifying others [12]. The Big Five Model embraces the idea that there are five core personality traits: neuroticism, extraversion, conscientiousness, agreeableness, and openness. Various instruments have been developed to measure these personality traits [13].

Contrary to views suggesting that personality traits are unchangeable [14], there are also new theories suggesting that personality can change as a result of certain life events and psychosocial processes [15,16].

Personality traits, traditionally considered stable over time, have been shown to exhibit flexibility under certain conditions. The distinction between **state effects** and **scar effects** offers a nuanced understanding of how temporary or long-lasting changes can occur: These refer to temporary changes in personality traits due to current emotional or mental states. For instance, anxiety and depression can cause short-term increases in neuroticism or decreases in extraversion and conscientiousness, which may revert once the individual’s mental state stabilizes [17]. In contrast, scar effects suggest that severe or prolonged psychological distress can leave lasting changes on personality traits, persisting even after recovery from the mental health episode. This can be particularly evident in traits like neuroticism, where repeated or chronic stress may cause long-term elevations [18].

Biological factors, including genetic predispositions and neurobiological mechanisms, play a critical role in personality trait stability and change. Twin studies have shown that while genetic factors significantly influence personality traits, the environment plays a crucial role in their development and modulation over time [19]. The interaction of neurotransmitters such as serotonin and dopamine with personality traits like neuroticism and extraversion has been explored, suggesting that changes in neurotransmitter levels due to stress or pharmacological interventions can alter these traits (20).

Environmental factors, including life experiences and social contexts, are pivotal in shaping and reshaping personality traits:

Significant life events, such as trauma or major life transitions, have been linked to changes in personality. For example, chronic job insecurity has been associated with increased neuroticism and decreased agreeableness and conscientiousness [20].

Positive social environments and supportive relationships can buffer the negative impact of stress on personality, facilitating resilience and positive personality changes [21].

In summary, while personality traits exhibit a degree of stability, they are also susceptible to changes influenced by both internal biological factors and external environmental conditions. Understanding the interplay between these factors and distinguishing between state and scar effects provides a comprehensive view of personality dynamics in response to psychological distress.

Besides the studies showing that personality traits are associated with the risk of anxiety and depressive disorders [19] there are also studies in the literature showing that anxiety and depressive symptoms also affect the personality traits temporarily (“state” effects) [17,18,22] or more permanently (“scar” effects). Anxiety and depressive disorders can generate “state” effects by altering individuals’ perception of personality traits [22]. Karsten et al. [17]. researched the state effects of depression and anxiety on the Big Five personality traits in adults. They reported evidence of state effects for increases in neuroticism in both depressive and anxiety disorders and decreases in extraversion and conscientiousness in depressive disorder. Another study reported that patients with symptoms of panic disorder and agoraphobia improved with changes in their personality (decrease in neuroticism) during treatment [23].

Medical students are particularly vulnerable to psychological distress [24]. The psychological and mental well-being of medical students is an important public health problem worldwide because it will affect the health care they will provide in the future [25,26]. Besides depression, many studies have reported that the prevalence of anxiety symptoms is high in medical students [1]. Personality traits play both direct and mediating roles in the well-being and success of medical students [27–31]. The literature has shown that personality traits, particularly extraversion and conscientiousness, are the characteristics most consistently associated with the academic performance of students [32,33]. Many studies have reported that individuals with major depressive disorder have lower extraversion and conscientiousness scores and higher neuroticism scores. A higher neuroticism score has been associated with a higher likelihood of suicide attempts and the persistence of depressive symptoms despite taking antidepressants [34]. Therefore, modifiable issues regarding factors predicting psychopathology should be emphasized. Personality traits may be enduring in nature but can change with experiences throughout life.

Some personality traits have been shown to be affected by depressive disorder status, but the situational effects of anxiety disorders on personality have been much less studied. In our study, we aimed to test whether there are changes in the Five Factor Model personality traits in response to anxiety and depression in medical students. We hypothesized that depression and anxiety would be high in medical students and that personality perception would be affected by depression and anxiety.

## Methods

We collected data using a Microsoft Forms questionnaire distributed to students via e-mail followed by a reminder and self-administered. The Ethics Committee of our Faculty of Medicine approved the research (7/91–2023). The study data were collected between 1 November 2023 and 1 May 2024. All participants were asked to sign a written informed consent form in pdf format along with the questionnaire and signed written informed consent forms. Participants were also asked to write their name, surname, and contact information on the sociodemographic data form to access information to identify individual participants during or after data collection.

### Procedure

A cluster sampling procedure was used to generate a representative sample of medical school students in grades 1st to 6th. We determined the sample size by the G*power (version 3.1.9.7) power analysis program. It was determined as 484 with a 95% confidence interval and a 5% margin of error, according to a reference study evaluating the relationship of personality traits with anxiety and depression [35].

Considering the possibility of them not attending, we invited via e-mail a total of 900 students in 6 classes, 150 students from each class. 800 students filled out the questionnaires. We also excluded those with missing data. We analyzed the data of 733 students. 416 students were in preclinical grades and 317 were in clinical grades.

The data of 733 students who filled out the questionnaires and scales between November 2023 and May 2024 were analyzed.

### Inclusion criteria

- 18 years or older,
- Still receiving preclinical/clinical training,
- Volunteering to participate.

### Exclusion criteria

-Younger than 18 years old,
-Not giving consent for participation
-With missing data.

### Measures

An assessment tool consisting of four sections was used: demographic information, screening for anxiety and depression with the Beck Anxiety Inventory (BAI) and the Beck Depression Inventory (BDI) respectively, and assessment of personality with the Five Factor Personality Inventory (FFPI).

### Sociodemographic data

A sociodemographic data form was developed by the authors to evaluate the sociodemographic characteristics. The data was prepared by the literature for evaluating the age, gender, marital status, school grade, income, residence area, smoking, alcohol use, suicide history, psychiatric history, and psychiatric history of the family.

### Five Factor Personality Inventory

It was developed by Benet-Martinez and John [13]. The Five Factor Personality Inventory consists of 44 items and is divided into Five different subscales: neuroticism, extroversion, conscientiousness, agreeableness, and openness. High scores obtained in the subscales mean that the participant has that personality trait at a high level. Five-Factor Personality Inventory is a 5-point Likert-type scale. The Turkish validity and reliability study was conducted [36]. In the current study, Cronbach’s alpha coefficients were found as follows: extraversion *α* =.71, neuroticism *α* =.73, agreeableness *α* =.56, conscientiousness *α* =.68, and openness *α* =.72.

### Beck Depression Inventory (BDI)

It is a self-administered scale to screen patients for depression risk and measure the level of depressive symptoms. It is a scale consisting of a total of 21 questions and 4-point Likert type items. It was developed by Beck et al. [37]. Each item receives increasing scores between 0–3 (0-not at all, 3-severe level). Below 10 points are considered normal, between 10–16 are considered mild depressive symptoms, between 17–29 are considered moderate, and 30 and above are considered severe depression. Turkish validity and reliability were determined by Hisli et al. [38]. Cronbach’s alpha coefficient was found to be.87 in this study.

### Beck Anxiety Inventory (BAI)

It was developed by Beck et al. [39] to determine the anxiety levels of individuals. The Turkish validity and reliability study was conducted by Ulusoy et al. [40]. The scale measures the level of anxiety symptoms. It includes a total of 21 items consisting of 4-point Likert type items. Each question receives a score between 0–3 (0-not at all, 3-serious level). The cut-off values used to diagnose anxiety are 0–7 points for normal, 8–15 points for mild anxiety, 16–25 points for moderate anxiety, and 26 and above for severe anxiety. Cronbach’s alpha coefficient was found to be.81 in this study.

### Statistical Analysis

Statistical analyzes were performed using IBM SPSS 20 analysis program. Data were presented as percentage, number, mean and standard deviation. Normal distribution of continuous variables was evaluated using Shapiro-Wilk and Kolmogorov Smirnov tests. Independent Sample t test was used for comparisons between two independent groups. In the correlation analysis of two continuous variables, the Pearson test was used for variables with normal distribution, and the Spearman test was used for variables that did not show normal distribution. Predicted risk factors between groups in multivariate analysis were analyzed using linear (Enter Model) regression analysis using possible risk factors identified in previous results. Statistical significance level was evaluated as p<0.05.

## Results

We analyzed the data of 733 participants including 297(40.5%) men and 436(59.5%) women in the study. The mean age was 22.03±2.54. 723(98.6%) were single, 723(98.6%) had a medium income rate, 161(22%) were smoking, 77(10.5%) were using alcohol, 642(87.6%) were living in a city center, 88 (12%) had a previous psychiatric history, 106(14.5%) had a previous psychiatric history of family, 19(2.6%) had a suicide history.

We re-grouped the participants into anxiety-non-anxiety and depression-non-depression groups. We determined those who scored 17 points and above in BDI as depression and those who scored 16 points and above in BAI as anxiety group, then compared the sociodemographic and clinical characteristics among each other. Accordingly, 233(31.8%) participants were evaluated as anxiety and 184(25.1%) participants as depression.

The mean age of the anxiety group was 21.89±2.20 and younger than the non-anxiety group (p=.028), the mean age of the depression group was 22.16±2.41 and the mean age of the non-depression group was 22.16±2.53. The rate of women was significantly higher in both groups (p for anxiety (pA)=.001, p for depression (pD)=.045). Those in the anxiety group tended to be at lower grades (p=.003) and were more likely to live in urban centers (p=.045). While the smoking rate was not different in the anxiety group compared to the non-anxiety group, it was higher in the depression group than in the non-depression group (pD=.003). Psychiatric history (pA=.000, pD=.000) and suicide history (pA=.007, pD=.000) were significantly higher in both diagnosed groups and psychiatric history of the family was also significantly higher for both (pA=.018, pD=.000).

BDI scores of the anxiety group were higher than the non-anxiety group (p=.000) and BAI scores of the depression group were also higher than the non-depression group (p=.000).

When the Big Five personality traits were evaluated; in the depression group neuroticism was significantly higher than in the non-depression group (p=.000), while extroversion (p=.004), conscientiousness (p=.000), agreeableness (p=.003), and openness (p=.001) were higher in the non-depression group. In the anxiety group, neuroticism was significantly higher (p=.000) than in the non-anxiety group while extroversion (p=.031), conscientiousness (p=.012), agreeableness (p=.028), and openness (p=.009) were higher in the non-anxiety group.

The sociodemographic and clinical characteristics of the participants are illustrated in Table 1.

**Table 1:** The sociodemographic and clinical characteristics of the participants.

	All Participants n=733	Anxiety n=233, 31.8%	p	Depression n=184, 25.1%	p
**Age**	22.03±2.54	21.89±2.20	.028*	22.16±2.41	.976
**Gender**					.045*
Women	436(59.5%)	158(67.8%)	.001^**^	121(65.8%)	
Men	297(40.5%)	75(32.2%)		63(34.2%)	
**Marital status**					.708
Single	723(98.6%)	232(99.6%)	.136	182(98.9%)	
Married	10(1.4%)	1(0.4%)		2(1.1%)	
**Income**					.002^**^
Low	55 (7.5%)	21(9%)	.065	21(11.4%)	
Medium	605(82.5%)	197(84.5%)		155(84.2%)	
High	73(10.5%)	15 (6.4%)		8(4.3%)	
**School Grade**					.400
1	49 (6.7%)	14(6.0%)		9(4.9%)	
2	167(22.8%)	72(30.9%)	.003^**^	53(28.8%)	
3	200(27.3%)	67(28.8%)		44(23.9%)	
4	80 (10.9%)	28(12.0%)		23(12.5%)	
5	225(30.7%	51(21.9%)		54(29.3%)	
6	12 (1.6%)	1(0.4%)		1(0.5%)	
**Residence area**					.151
-Village	46 (6.3%)	21(7.7%)	.045*	17(9.2%)	
-Town	45 (6.1%)	18 (9.0%)		10(5.4%)	
-City	642(87.6%)	194 (83.3%)		157(85.3%)	
**Smoking**					.003^**^
Yes	161(22%)	53(22.7%)	.727	55(29.9%)	
No	572(78%)	180(73.3%)		129(70.1%)	
**Alcohol**					.308
Yes	77(10.5%)	25(10.7%)	.892	23(12.5%)	
No	656(89.5%)	208(89.3%)		161(87.5%)	
**Suicide history**					.000^***^
Yes	19(2.6%)	12(5.6%)	.007^**^	13(7.07%)	
No	714(97.4%)	221(94.4%)		171(92.93%)	
**Psychiatric history**					.000^***^
Yes	88 (12%)	49(21.03%)	.000^***^	43(23.4%)	
No	645(88%)	184(78.97%)		141(76.6%)	
**Psychiatric history** **of family**					.000^***^
Yes	106(14.5%)	47(20.2%)	.018^**^	42(22.8%)	
No	727(85.5%)	186(79.8%)		142(77.2%)	
**BAI total score**	15.29±13.10	30.14±9.84	.000^***^	27.23±13.70	.000^***^
**BDI total score**	14.20±9.89	20.54±10.37	.000^***^	26.60±8.50	.000^***^
**neuroticism**	24.89±6.27	27.00±6.3	.000^***^	28.32±5.94	.000^***^
**extroversion**	25.65±5.51	25.07±5.35	.031*	24.73±5.05	.004^**^
**conscientiousness**	30.93±5.234	30.26±4.83	.012*	29.70±5.13	.000^***^
**agreeableness**	31.40±4.921	31.12±4.57	.028*	30.78±5.03	.003^**^
**openness**	33.97±6.44	33.26±6.51	.009^**^	32.81±6.63	.001^**^

*p <.05;

** p <.01;

*** p <.001

Anxiety group is compared to non-Anxiety group and Depression Group is compared to non-Depression group

BAI: Beck Anxiety Inventory, BDI: Beck Depression Inventory

When we evaluated the correlations between the Big Five personality traits and anxiety; neuroticism was positively correlated with BAI scores (r=.325, p=.000) while extroversion (r=-.149, p=.000), conscientiousness (r=-.184, p=.000), agreeableness (r=-.154,p=.000), and openness (r=-.173, p=.000) were negatively correlated with BAI scores.

When we evaluated the correlations between the Big Five personality traits and depression, it was found that neuroticism was positively correlated with BDI scores (r=.469, p=.000) while extroversion(r=-.185, p=.000), conscientiousness (r=-.211, p=.000), agreeableness (r=-.172, p=.000), and openness (r=-.167, p=.000) traits were negatively correlated with BDI scores.

When correlations were examined according to gender and psychiatric history, the correlation of depression and anxiety scores was found to be consistent with the entire group in both women and in the group without psychiatric history, while depression scores were found to be related to neuroticism and anxiety scores to neuroticism and openness in men and in groups with a positive psychiatric history.

The correlations between the Big Five personality traits and Anxiety and Depression scores are illustrated in Table 2.

**Table 2:** The correlations between the Big five personality traits and Anxiety and Depression scores.

Characteristics		BAI total	BDI total	extroversion	conscientiousness	agreeableness	openness	neuroticism	age	Income	school grades
BAI total	r	**1**	**.579** ^**^	**-.149** ^**^	**-.184** ^**^	**-.154** ^**^	**-.173** ^**^	**.325** ^**^	-.082*	**-.112** ^**^	**-.173** ^**^
	p		**.000**	**.000**	**.000**	**.000**	**.000**	**.000**	**.039**	**.005**	**.000**
BDI total	r	**.579** ^**^	**1**	**-.185** ^**^	**-.211** ^**^	**-.172** ^**^	**-.167** ^**^	**.469** ^**^	-.016	**-.186** ^**^	**-.107** ^**^
	p	**.000**		**.000**	**.000**	**.000**	**.000**	**.000**	.677	**.000**	**.004**
Extroversion	r	**-.149** ^**^	**-.185** ^**^	**1**	**.157** ^**^	**.242** ^**^	**.320** ^**^	**-.192** ^**^	.014	.081*	-.032
	p	**.000**	**.000**		**.000**	**.000**	**.000**	**.000**	.712	**.032**	.384
Conscientiousness	r	**-.184** ^**^	**-.211** ^**^	**.157** ^**^	**1**	**.357** ^**^	**.183** ^**^	**-.234** ^**^	-.037	.046	**-.126** ^**^
	p	**.000**	**.000**	**.000**		**.000**	**.000**	**.000**	.318	.226	**.001**
Agreeableness	r	**-.154** ^**^	**-.172** ^**^	**.242** ^**^	**.357** ^**^	**1**	**.242** ^**^	**-.250** ^**^	.003	-.029	-.020
	p	**.000**	**.000**	**.000**	**.000**		**.000**	**.000**	.943	.433	.590
Openness	r	**-.173** ^**^	**-.167** ^**^	**.320** ^**^	**.183** ^**^	**.242** ^**^	**1**	**-.120** ^**^	-.006	-.025	-.056
	p	**.000**	**.000**	**.000**	**.000**	**.000**		**.001**	.867	.494	.129
neuroticism	r	**.325** ^**^	**.469** ^**^	**-.192** ^**^	**-.234** ^**^	**-.250** ^**^	**-.120** ^**^	**1**	-.019	-.033	.009
	p	**.000**	**.000**	**.000**	**.000**	**.000**	**.001**		.616	.385	.818

*p <.05;

** p <.01;

*** p <.001

BAI: Beck Anxiety Inventory BDI: Beck Depression Inventory

After adjusting for control variables such as age, gender, income, school grades, and psychiatric and suicide history, both the neuroticism and openness models remained significant. In the neuroticism model, BAI total (indicative of anxiety symptoms), BDI total (indicative of depressive symptoms), extroversion, conscientiousness, and agreeableness continued to show significant associations, while female gender, younger age, and psychiatric disorders were identified as confounding factors. Similarly, in the openness model, BAI total (indicative of anxiety symptoms), agreeableness, and extroversion remained significant, with older age and school grades acting as confounding factors. These findings suggest that personality traits interact with both emotional symptoms and demographic variables, highlighting the complex influences on neuroticism and openness.

**Fig 1:**
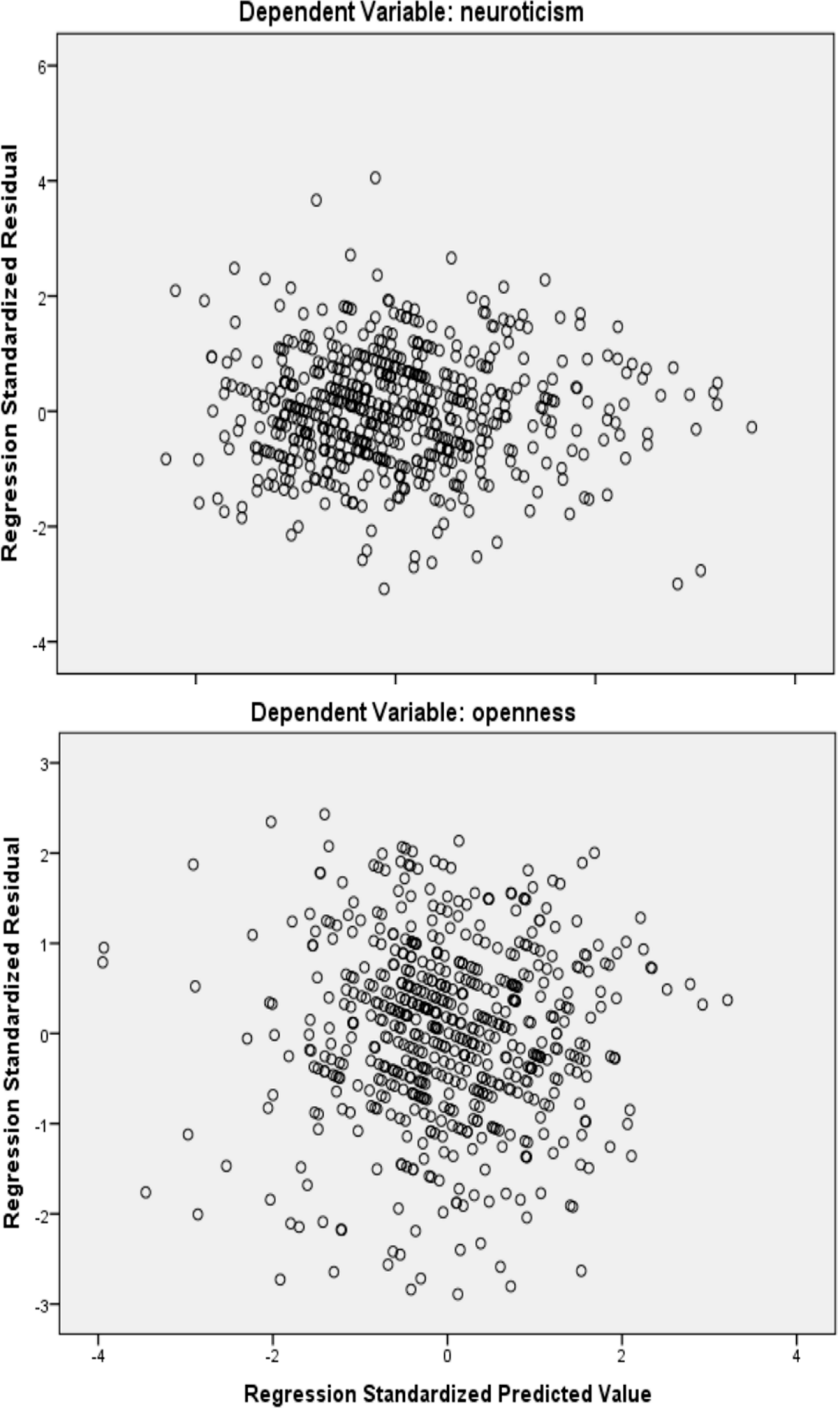
Linear Regression Analysis of neuroticism and openness personality traits.

Linear Regression Analysis of the Big Five personality traits is illustrated in Table 3 and Fig 1.

**Table 3:** Linear Regression Analysis of neuroticism and openness personality traits.

Model	Unstandardized Coefficients		Standardized Coefficients		t	Sig.	Correlations				Collinearity Statistics	
	B		Std.Error	Beta			Zero-order	partial	part		tolerence	VIF
**Dependent variable neuroticism**												
1 (Constant)	30.547	2.126			14.369		.000					
BAI total	.026	.021	.055		3.248		.**001**	.326	.052	.044	.647	1.545
BDI total	.251	.028	.397		8.940		**.000**	.479	.347	.316	.633	1.579
Extroversion	-.090	.044	-.079		-2.073		**.039**	-.192	-.085	-.073	.858	1.166
Conscientiousness	-.101	.046	-.085		-2.197		**.028**	-.232	-.090	-.078	.840	1.190
agreeableness	-.160	.051	-.123		-3.164		**.002**	-.245	-.130	-.112	.822	1.217
Openness	.026	.037	.026		.686		.493	-.120	.028	.024	.856	1.169
1 (Constant)	30.547	2.126			14.369		**.000**					
BAI total	.068	.022	.146		3.028		**.001**	.387	.141	.111	.576	1.737
BDI total	.123	.026	.226		4.708		**.000**	.438	.216	.173	.582	1.718
Extroversion	-.123	.043	-.117		-2.847		**.008**	-.226	-.133	-.104	.798	1.253
Conscientiousness	-.202	.045	-.180		-4.487		**.000**	-.330	-.206	-.165	.834	1.198
agreeableness	-.172	.050	-.140		-3.425		**.005**	-.276	-.159	-.126	.807	1.240
Openness	.001	.044	.001		.034		.973	-.091	.002	.001	.776	1.288
Age	-.426	.171	-.144		-2.492		**.013**	-.088	-.103	-.081	.428	2.335
Schoolgrades	.491	.254	.109		1.934		.054	-.003	.091	.071	.422	2.312
Suicide history	1.472	1.299	.046		1.134		.257	.197	.053	.042	.812	1.232
psychiatric history	2.170	.657	.140		3.303		**.002**	.298	.153	.121	.748	1.338
Income	.593	.688	.032		.861		.389	-.001	.040	.032	.952	1.051
**Dependent variable openness**												
1 (Constant)	18.621	2.638			7.060		.000					
BAI total	-.049	.023	-.100		-2.109		**.035**	-.182	-.087	-.081	.650	1.538
BDI total	-.029	.033	-.045		-.877		.381	-.172	-.036	-.034	.558	1.792
conscientiousness	.089	.051	.073		1.744		.082	.179	.072	.067	.838	1.194
agreeableness	.164	.056	.123		2.917		**.004**	.226	.120	.111	.820	1.220
neuroticism	.032	.046	.031		.686		.493	-.120	.028	.026	.731	1.368
extroversion	.304	.047	.259		6.473		**.000**	.316	.259	.247	.913	1.096
1 (Constant)	18.621	2.638			7.060		**.000**					
BAI total	-.053	.024	-.121		-2.214		**.035**	-.126	-.103	-.092	.570	1.754
BDI total	.002	.029	.003		.062		.883	.034	.003	.003	.555	1.802
extroversion	.288	.045	.289		6.449		**.000**	.363	.290	.267	.856	1.168
conscientiousness	.099	.049	.093		1.946		.052	.170	.095	.084	.806	1.241
agreeablenes	.196	.054	.168		3.645		**.001**	.266	.169	.151	.809	1.236
neuroticism	.002	.050	.002		.034		.973	-.091	.002	.001	.609	1.641
Age	.469	.177	.167		2.651		**.007**	.002	.124	.110	.430	2.324
Schoolgrades	-.900	.270	-.211		-3.335		**.001**	-.124	-.155	-.138	.428	2.334
Suicide history	.906	1.393	.030		.651		.516	.066	.031	.027	.810	1.234
psychiatric history	1.361	.710	.093		1.919		.056	.107	.090	.079	.736	1.359
Income	.250	.738	.014		.338		.735	-.001	.016	.014	.951	1.052

BAI: Beck Anxiety Inventory BDI: Beck Depression Inventory

## Discussion

In this current study, the impact of anxiety and depression levels on personality perception in medical students was evaluated. Anxiety and depression symptoms were found with a prevalence of 31.8% and 25.1% respectively among medical students and were correlated with their personality traits. Increased levels of anxiety and depression were positively correlated with neuroticism and negatively correlated with extroversion, conscientiousness, agreeableness, and openness. Both depression and anxiety influenced neuroticism and anxiety influenced openness. This study highlights the impact of anxiety and depression on personality traits among medical students. While the results are consistent with existing literature, they also suggest the need for targeted interventions to reduce these effects. Personality traits, particularly neuroticism and openness, significantly altered the response to psychological distress, highlighting the importance of mental health support in medical education.

Previous literature is rich in studies on the impact of personality on anxiety, depression, and stress, but recent studies on the state effect of anxiety, depression, and stress on personality perception are limited [17,20,23].

Personality, although often assumed to be static, changes in various ways throughout life. Environmental influences and stressful life events trigger a change in personality by reshaping a person’s thoughts, feelings and behaviors [20].Among the Five factor personality traits, there is the most evidence that neuroticism may be the trait most responsive to stress. Neuroticism tends to increase when individuals experience a large amount of distress through an extremely aversive event or a depressive episode [21]. Neuroticism is a personality trait associated with both anxiety and depression [41]. In the longitudinal study of Prince et al., neuroticism increased in the patients who developed agoraphobia, GAD, first-onset panic disorder, or MDD during the follow-up period.

In the study of Karsten et al. [17]; While both anxiety and depressive disorders showed state effects on neuroticism, depressive disorders mainly affected extraversion and conscientiousness, and there were no state effects for agreeableness and openness.

In our study, neuroticism was associated with both depression and anxiety. It has been shown in the literature that medical students, especially those with neuroticism, are more prone to developing psychological distress and burnout. Neuroticism is often associated with a vulnerability to negative emotions and distress, pessimism, irritability, and poor coping ability. Personality traits are associated with many aspects for medical students, including academic performance [42].

Identifying and early intervention of anxiety and depressive symptoms that increase the level of neuroticism will prevent the increase in neuroticism, which affects academic performance such as learning, success, and mental well-being as well as the negative effects of depressive complaints.

In our study, anxiety particularly negatively affected openness. Openness to experience is one of the ‘Big Five’ personality traits and is generally associated with creativity, curiosity and intelligence [43]. The literature has shown that personality traits such as extraversion, conscientiousness, openness, and agreeableness play important roles in contributing to students’ academic success. Researchers noted that “Openness reflects the ideal student” because of its association with being resourceful, intelligent, and forward-thinking. In the Five Factor model, openness was positively associated with the learning motivation, approach to learning and critical thinking and negatively related to absenteeism [44].

In this study, both depression and anxiety levels were negatively correlated with extraversion, conscientiousness, agreeableness, and openness personality traits which are important determinants of academic success, and anxiety had especially negatively influenced openness.

The state-perhaps scar effect that anxiety and depression have on personality traits, as well as their symptoms, seems to have an additional impact on the academic performance of students. Studies are reporting that personality changes are permanent even after treatment [22]. Studies have found that neuroticism increases and extraversion decreases during depressive episodes, either temporarily (state effect) or persistently in some studies (scar effect), but not in all studies. While most studies have focused on changes in neuroticism and sometimes on changes in extraversion, less is known about changes in conscientiousness, agreeableness, or openness, although there are studies that have found these to be stable during a depressive episode. There is also limited evidence that neuroticism decreases and extraversion increases when anxiety symptoms improve in patients with depressive disorders [17].

Medical schools should implement programs such as stress reduction techniques to help medical students cope with their stress, increase resilience, and prevent psychopathology [38]. In addition, regular screening for conditions such as anxiety, depression, and stress will be beneficial for medical faculties as it will help identify the medical students at risk and conduct programs that support their personal development.

## Limitations and strengths of the study

Our study has some limitations. Firstly, since it is a cross-sectional study, it is not possible to draw causal relationships. The findings of this study require to be supported by future prospective cohort studies. The second limitation is that the data were obtained through self-reported surveys, which could lead to response bias. Considering that the study sample was medical students, it may not be appropriate to generalize the results. More research should be conducted in other schools and cultures. In addition to its limitations, it also has some strengths. Studies on the effects of anxiety and depression on personality perception are limited, and in this sense, it can be said that the study contributes to the literature, and the sample size can be considered satisfactory.

## Conclusion

In conclusion, neuroticism and openness appear to covary with mood changes. Both depressison and anxiety had an impact on neuroticism, and anxiety had an impact on openness. Our study supports the existing literature on this topic. However, these results should be confirmed with prospective cohort studies.

## Supporting information

S1 File SPSS data information for the research. (SAV)
